# Synergetic effects of silicon nanoparticles and multi-walled carbon nanotubes on the structural and dielectric properties of polystyrene-based nanocomposites

**DOI:** 10.3389/fchem.2026.1789689

**Published:** 2026-04-24

**Authors:** María Fernanda Cuenca-Lozano, Habiba Aslan Shirinova, Lala Rasim Gahramanli, Flora Vidadi Hajiyeva, Sevinj Garib Nuriyeva, Aynura Hidayat Karimova, Eldar Kochari Gasimov, Fuad Huseynali Rzayev, Aysel Ilgar Masimova, Narmin Yashar Suleymanova, Raida Zabit Ibaeva, Cristian Vacacela Gomez

**Affiliations:** 1 Departamento de Producción, Facultad de Ciencias Exactas y Naturales, Universidad Técnica Particular de Loja, Loja, Ecuador; 2 Department of Chemical Physics of the Nanomaterials, Baku State University, Baku, Azerbaijan; 3 Nanoresearch Laboratory, Baku State University, Baku, Azerbaijan; 4 Faculty of Chemistry, Baku State University, Baku, Azerbaijan; 5 Department of Cytology, Embryology and Histology, Azerbaijan Medical University. Nasimi reg., Baku, Azerbaijan; 6 Department of Electron Microscopy, Scientific Research Center, Azerbaijan Medical University. Nasimi reg., Baku, Azerbaijan; 7 Sumgayit State University, Sumgayit, Azerbaijan; 8 Institute of Physics, Ministry of Science and Education of the Republic of Azerbaijan, Baku, Azerbaijan; 9 INFN-Laboratori Nazionali di Frascati, Frascati, Rome, Italy; 10 Universidad Ecotec, Samborondón, Ecuador

**Keywords:** HIPS, hybrid composite, interfacial polarization, multi-walled carbon nanotubes, nano- silicon

## Abstract

In the present study, high-impact polystyrene (HIPS) nanocomposites filled with nano-silicon In the present study, high-impact polystyrene (HIPS) nanocomposites filled with nano-silicon particles (Si) and multi-walled carbon nanotubes (MWCNTs) were prepared, their structural, dielectric, and mechanical properties were investigated. X-ray diffraction (XRD) analysis confirmed the polycrystalline nature of Si nanoparticles and the preservation of the MWCNTs’graphitic (002) structure. Atomic Force Microscope (AFM) analysis confirmed that the addition of hybrid fillers leads to changes in the surface morphology. It was determined that the root mean square (RMS) surface roughness decreased from approximately 85 nm for pure MWCNTs to about 30 nm for the HIPS + Si + MWCNT nanocomposite. Dielectric measurements showed that the HIPS+1.5 wt.%Si+1.5 wt.%MWCNT nanocomposite exhibited the highest dielectric response. The dielectric constant increased by approximately 3.5 times at 1 kHz compared to neat HIPS. A relaxation maximum observed at 125 Hz and was corresponding to an effective relaxation time of τ = 1.27 m. At 200 kHz, the AC conductivity was calculated as σ ≈ 10^–8^ S/cm, consistent with near-percolation behavior. Mechanical testing revealed that tensile strength increased from 14.06 MPa to 17.49 MPa with hybrid filler incorporation, while relative elongation decreased from 82% to 17.88%, indicating the typical stiffness–ductility trade-off. The results demonstrate that hybrid Si/MWCNT fillers induce synergistic effect on dielectric and mechanical performance in HIPS-based nanocomposites.

## 1 Introduction

Silicon (Si) is one of the key components of microelectronics (Baca-Arroyo et al., 2021; Kamal, 2022). Among the primary uses for silicon are microchips and semiconductor device manufacturing, including solar cells, portable computer displays, and other applications (Li et al., 2024; Offrein et al., 2018; Prishya et al., 2022). Unlike bulk silicon, nano-silicon (n-Si) exhibits unique optical and electrical properties, further expanding its traditional application areas. The use of n-Si as an anode material, especially in lithium-ion batteries (LIBs), has been the subject of numerous investigations (Lai et al., 2020; Lu et al., 2022; Wang et al., 2024). n-Si possesses a high theoretical specific capacity (4200 mAhg^1^), making it one of the most desirable anode-active materials for LIBs. Another promising area of exploration for n-Si-based materials is the advancement of photonic crystals (Ondič et al., 2017; Paul et al., 2018). They are also desirable options for biological applications due to their chemical stability and biocompatibility (Fu et al., 2025; He et al., 2018; Premnath et al., 2015).

Notwithstanding these encouraging characteristics, issues including significant volume expansion, mechanical instability, and restricted electrical conductivity during repeated charge–discharge or temperature cycles frequently impede the practical application of n-Si-based nanomaterials. To get over these restrictions, current studies have concentrated more on creating hybrid nanosystems that mix n-Si with other nanostructured materials (Shirinova et al., 2025; Zhang et al., 2018).

Each component can complement the others thanks to these synergistic pairings, producing materials with markedly improved mechanical, electrical, thermal, and chemical stability. Hybrid nanomaterials are gaining popularity due to their potential uses in biotechnology, electronics, energy, catalysis, and electromagnetic wave control. The idea of “one material–multiple functionalities” is embodied by these materials. This paradigm creates new avenues for managing structure-property correlations at the nanoscale and pushes the limits of traditional materials. Furthermore, improving phase dispersion and fortifying interfacial contacts are thought to be crucial elements in the development of next-generation, flexible, high-performance functional materials (Gahramanli et al., 2025; Gulahmadov et al., 2025; Hajiyeva et al., 2025b; Lin et al., 2018; Ramazanov et al., 2021b).

Various hybrid materials with silicon, such as n-Si-Au (Fang et al., 2022), Fe-Si (Bernhard et al., 2025), Si-SiC (Kshatri et al., 2022; Sheikhan and Narayanan, 2024), Si-MXENE (Liu et al., 2025), and Si-GO (Pachiyappan and Gnanasundaram, 2020) was produced and explored. Among various hybrid systems, n-Si and carbon nanotube (CNT) (Gong et al., 2024; Liang et al., 2025; Wang et al., 2025; Xiao et al., 2024; Xiao et al., 2026; Yao et al., 2025; Zhan et al., 2024; Zhan et al., 2025) composites have attracted significant attention due to their synergistic combination of high theoretical capacity from silicon and excellent electrical conductivity and mechanical resilience from carbon nanotubes.

It was demonstrated that the magnetron sputter-fabricated Si-coated MWCNT tissue composite material may function as a flexible, freestanding anode for LIBs. In comparison to the uncoated MWCNT tissue, this composite electrode showed a substantial increase in gravimetric capacity from 109 to 290 mAhg^1^ and a Coulombic efficiency of up to 94% following the first ten creation cycles. The resulting hybrid composite anode is a viable candidate for flexible LIB applications due to the straightforward and economical nature of the suggested synthesis technique (Huang et al., 2024).

A freestanding single-walled carbon nanotube (SWCNT) film was dry-transferred onto a silicon substrate to create a CNT/Si_3_N_4_/Si heterojunction photodetector. To provide a consistent photocurrent, the CNT film functioned as a transparent charge-collecting electrode. In the visible spectrum, the device showed a photocurrent that increased with wavelength and was linearly dependent on light intensity. At 640 nm, the external quantum efficiency reached 65% while remaining intensity-independent (Capista et al., 2022).

Despite the functional potential of Si–CNT hybrid nanostructures, their large-scale practical application is still restricted due to processing challenges and poor structural integrity. One practical way to get around these restrictions is to incorporate Si-CNT hybrid nanostructures into polymer matrices. A continuous matrix offered by polymers facilitates better hybrid nanostructure dispersion. Incorporating n-Si–MWCNT hybrid nanostructures into thermoplastic polymers appears to be a viable path toward multifunctional materials. Although the properties of Si–CNT hybrid systems have been investigated to some extent by several authors, the synthesis and study of polymer nanocomposites based on these hybrid systems remain highly relevant. In particular, the development of a fabrication method, as well as the investigation of the structure and properties of multifunctional nanocomposites based on widely used, recyclable thermoplastic polymers and n-Si-MWCNT hybrid systems, is of great importance both for fundamental science and for large-scale industrial applications (Shan et al., 2019; Siwal et al., 2020).

In the presented study, various percentages of Si and MWCNT were used as fillers in a wide range using high-impact polystyrene (HIPS) polymer. Solution melting and hot pressing methods were used to produce HIPS + Si + MWCNT nanocomposite, and the structure and dielectric properties of the nanocomposites were investigated depending on filler content.

## 2 Materials and methods

### 2.1 Preparation of the nanocomposites

Mixing in solution and hot pressing were used to create nanocomposites. Firstly, 0.5 g high-impact polystyrene granules were dissolved in 100 mL toluene solvent at 120 °C using a magnetic stirrer set at 700 rpm for approximately 30 min. Then, nano-Silicon powder in different amounts (1; 1,5; 3%) was slowly added to the dissolved polymer and intensively stirred on a magnetic stirrer for 1 h. The MWCNT with different mass amounts was dissolved in 20 mL toluene. The MWCNTs + toluene mixture was added to the dissolved polymer + nano-silicon system and mixed at the 110 °C for 1 h. The final HIPS + Si + MWCNT nanocomposite layers with 100 μm thickness are obtained from nanocomposite ingots at the 110 °C and a pressure of 10 MPa by the hot pressing method (Shirinova et al., 2024).

### 2.2 Research methods of polymer nanocomposites

X-ray diffraction was performed on the Rigaku Mini Flex 600 XRD diffractometer at ambient temperature. In all the cases, Cu Kα radiation from a Cu X-ray tube (run at 15 mA and 30 kV) was used. The samples were scanned in the 2θ angle range of 20°–70°.

The morphology of the samples was investigated by the Transmission Electron Microscope (TEM) JEM-1400 (JEOL, Japan) at a voltage of 80–120 kV. Samples were prepared by a drop-dry method on carbon-coated 200-mesh copper grids (Electron Microscopy Science, USA). Morphometric analysis of the nanoparticles electronograms was performed in TIF format using the TEM Imaging Platform software version number 5.2 (Build 3554) developed by Olympus Soft Imaging Solutions GmbH (Münster, Germany).

SEM analysis of nanocomposites was performed on a Jeol-JSM 7600 F scanning electron microscope. The accelerating voltage is 15 kV, the working distance = 4.5 mm.

Atomic Force Microscopy (AFM) measurements were performed using an NT-MDT Integra Prima (Zelenograd, Russia) AFM operating in tapping mode. The surface topography and roughness of nanocomposite films were assessed in non-contact mode under ambient conditions.

The dielectric permittivity and dielectric loss of the nanocomposites were measured using an MNIPI E7-20 immittance meter. The frequency dependence of capacitance and dielectric loss was recorded at a temperature of 293 K in the frequency range of 25 Hz to 1 MHz. For dielectric measurements, three specimens with the same composition and concentration were prepared. At each frequency, the reported values represent the average of the measurements. The measurement uncertainty did not exceed ±5%.

Tensile test specimens were prepared in accordance with ASTM D638-2003 using a standard dog-bone geometry with an overall length of 110 mm. Tensile tests were carried out using a universal testing machine at room temperature until fracture. For each composition, the tensile measurements were performed on four different specimens to ensure reproducibility. The elongation at break was recorded, and the tensile strength at break (MPa) was calculated from the applied force and the cross-sectional area. The reported values represent the average of these measurements.

## 3 Results and discussion

**FIGURE 1:**
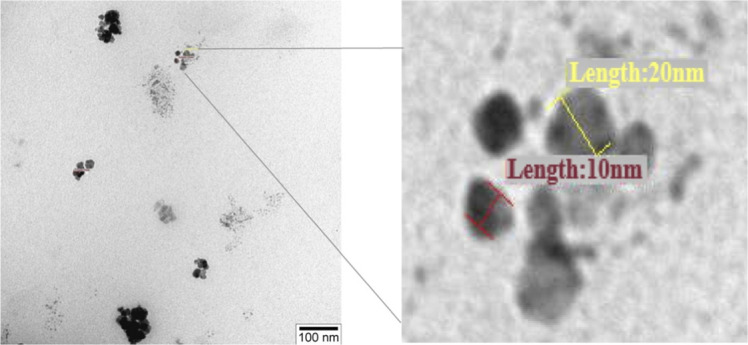
TEM images of the Silicon nanoparticles.

The TEM images of the pure Si nanoparticles are displayed in Figure 1. The size range of the Si nanoparticles, approximately 10–20 nm, is confirmed by the TEM investigation, which also confirms their nanoscale dimensions and relatively homogeneous shape.

**FIGURE 2:**
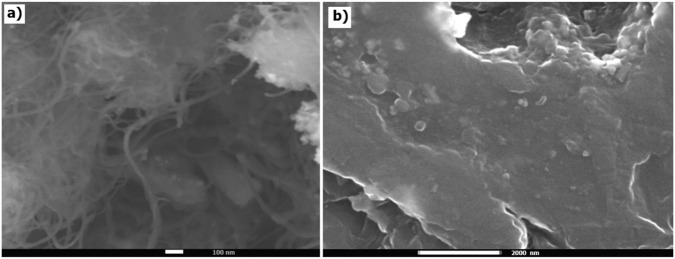
SEM images of **(a)** the pure MWCNT and **(b)** PS + Si nanocomposite.

Figure 2a shows the SEM image of pure MWCNTs. The nanotubes have an average diameter of approximately 10–20 nm. The SEM micrograph reveals a highly entangled and interconnected tubular morphology characteristic of multi-walled carbon nanotubes. The nanotubes are randomly oriented and form a network-like structure. The SEM image of the PS + Si nanocomposite shows a relatively continuous matrix with a heterogeneous surface morphology (Figure 2b).

**FIGURE 3:**
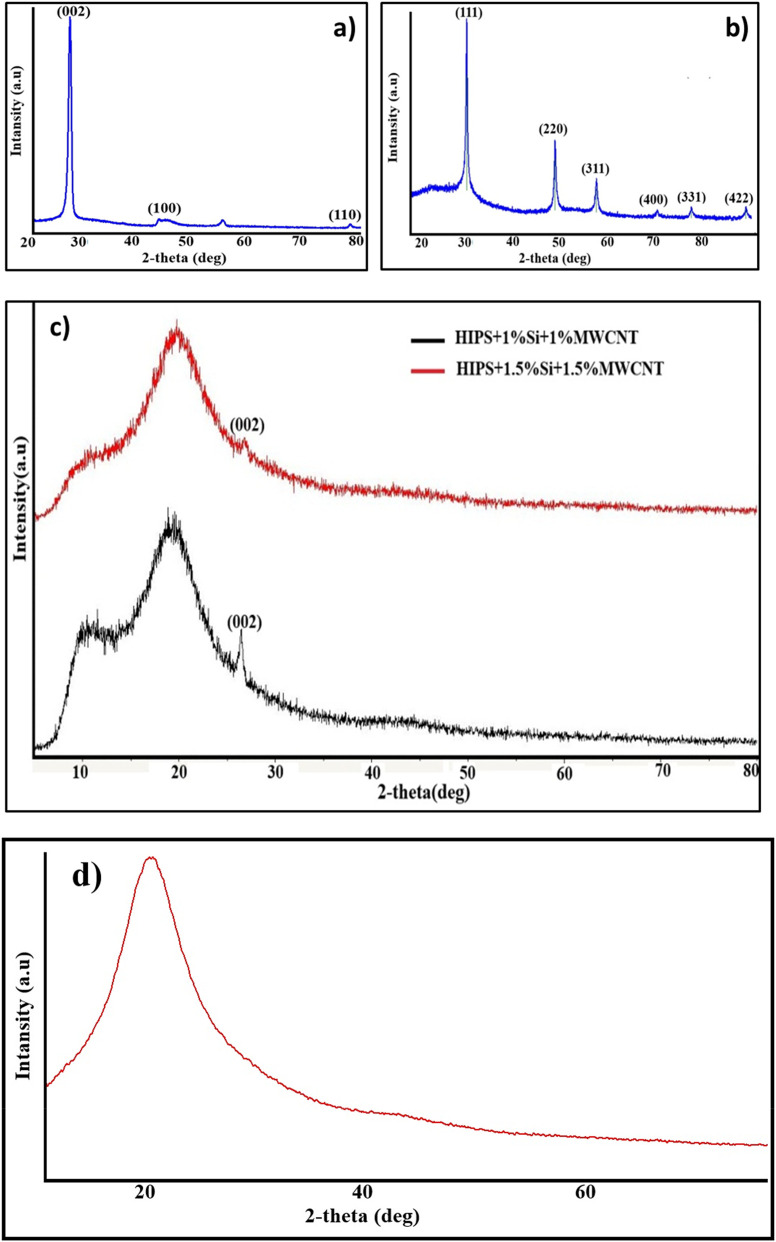
XRD pattern of the **(a)** pure MWCNT and **(b)** Si nanoparticles **(c)** HIPS + Si + MWCNT nanocomposites **(d)** pure PS.

The pure MWCNT’s X-ray diffraction (XRD) pattern is shown in Figure 3. The graphitic structure of carbon in the MWCNTs is responsible for the distinctive peaks seen at 2θ = 26° and 43°, as depicted in the image, which corresponds to file No. 98-008-5678 from the PAN-ICSD Reference Database (Hajiyeva et al., 2024). The weak diffraction peak observed in the 55°–60° region can be assigned to the (004) reflection of graphitic MWCNTs (Bajorek et al., 2022).

The XRD pattern of Si nanoparticles is shown in Figure 2b. The diffraction peaks observed at 2θ values corresponding to the (111), (220), (311), (400), (331), and (422) planes are characteristic of crystalline silicon and are consistent with the standard cubic diamond structure (JCPDS). The presence of multiple sharp peaks indicates that the Si nanoparticles exhibit a polycrystalline nature (Shirinova et al., 2024). Figure 2c demonstrates the XRD pattern of the HIPS + Si + MWCNT nanocomposite. Analysis confirms that nanocomposites exhibit an amorphous structure characteristic of the HIPS matrix (Figure 2d). The (002) plane of MWCNT is represented by the diffraction peak at 2θ = 26°, which validates the preservation of the graphitic structure within the polymer matrix. XRD analysis shows that increasing the MWCNT content from 1% to 1.5% leads to a slight decrease in the intensity of the (002) peak. Rubel et al. (2019) reported a similar observation and attributed it to the desegregation of nanotube bundles, indicated by the disappearance of lattice peaks associated with the bundled structure (Rubel et al., 2019). The low concentration and efficient dispersion of Si nanoparticles are responsible for their lack of identifiable crystalline peaks, as commonly reported (Mendoza-Duarte et al., 2024; Wahab et al., 2020; Zhang et al., 2025). The amorphous halo becomes even wider as the amount of nanofiller increases. This suggests that the ordering of the polymer chain is more disrupted.

**FIGURE 4:**
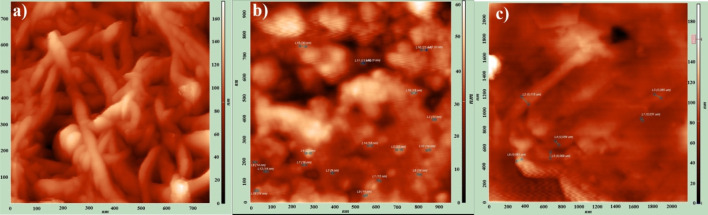
AFM -2D images of the **(a)** pure MWCNT and **(b)** HIPS + Si nanoparticles **(c)** HIPS + Si + MWCNT nanocomposites.

**FIGURE 5:**
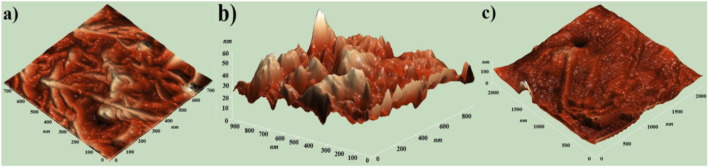
AFM -3D images of the **(a)** pure MWCNT and **(b)** HIPS + Si nanocomposites **(c)** HIPS + Si + MWCNT nanocomposites.

The surface morphology of pure MWCNTs, HIPS + Si, and HIPS + Si + MWCNT nanocomposites was examined by atomic force microscopy (AFM) in both 2D and 3D modes (Figures 4, 5).

In comparison to pure MWCNTs, the 2D AFM image of the HIPS + Si nanocomposites shows a smoother surface. The bright areas represent Si nanoparticles implanted in the HIPS matrix. Among all the samples, the HIPS + Si + MWCNT nanocomposite exhibits the most intricate surface morphology. A comparison of the three systems reveals that the addition of nanofillers significantly alters the HIPS’ surface morphology. As Kuzmenko et al. (2020) noted in their work, it is observed that MWCNT forms bent structures in our nanocomposites (Kuzmenko et al., 2020). However, in the HIPS + Si + MWCNT nanocomposite, Si and MWCNT form a hierarchical hybrid structure in the polymer. Mahmoudi et al. (2020) studied the morphology of PVC + Si + MWCNT nanocomposite using AFM and showed that interstitial cavities are formed in the polymer structure with the introduction of Si + MWCNT fillers. The formation of porous regions is also clearly visible in the 2D and 3D images of HIPS + Si + MWCNT nanocomposites (Mahmoudi et al., 2020). Si and MWCNT fillers in HIPS create a hierarchical hybrid morphology that results in unique surface features that directly affect the functional capabilities of the composite.

**FIGURE 6:**
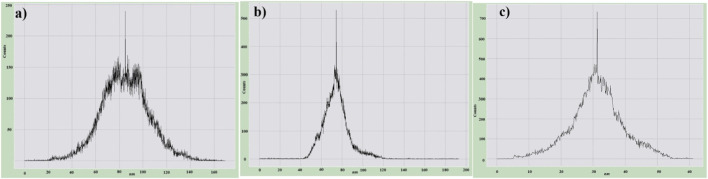
The surface roughness histograms of **(a)** pure MWCNT, **(b)** HIPS + Si, and **(c)** HIPS + Si + MWCNT nanocomposites.

Figure 6 demonstrates the surface roughness histograms of pure MWCNT, HIPS + Si, and HIPS + Si + MWCNT nanocomposites. Pure MWCNTs exhibit a height-distribution histogram of the surface root-mean-square (RMS) roughness that follows a Gaussian-like profile, centered at approximately 85 nm. For the HIPS + Si nanocomposite, the surface RMS roughness is about 70 nm. In contrast, for the ternary system HIPS + Si + MWCNT, the surface RMS roughness differs significantly from the values obtained for the other two nanocomposite samples. It was found that in the HIPS + Si + MWCNT system, refinement of the structural elements occurs, and the RMS roughness is around 30 nm. It should be noted that, for pure MWCNT, HIPS + Si, and HIPS + Si + MWCNT systems, the same tendency was observed in measurements carried out at different regions of the samples. In particular, the RMS roughness of the pure MWCNT surface is higher than that of the HIPS + Si + MWCNT system. This is related to the fact that, unlike pure MWCNT, in the HIPS + Si + MWCNT system the bundled structure of MWCNTs disappears, and due to dispersion, the nanotubes become distributed throughout the polymer volume. AFM images similar to those observed in our study, as well as comparable results, have also been reported by other authors (Vidali et al., 2015). It should also be noted that the changes associated with the dispersion of MWCNT nanoparticles in the polymer and the elimination of the bundled structure are consistent with the XRD results.Similarly, the lower RMS roughness of the HIPS + Si + MWCNT system compared to HIPS + Si is related to the surface structure being more strongly governed by the polymer phase morphology in the latter system, where the filler content is lower. In the ternary system, the higher content of the filler phase leads to the refinement of the surface features (Kesarwani et al., 2023).

**FIGURE 7:**
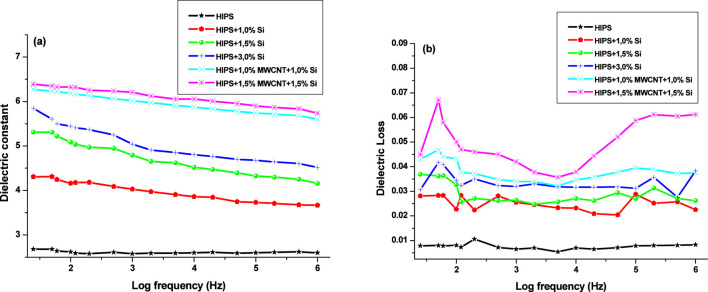
Dielectric properties of HIPS-based polymer nanocomposites **(a)** dielectric permittivity; **(b)** the dielectric loss.

The dielectric properties of HIPS + Si + MWCNT nanocomposites were comparatively analyzed. Figure 7 illustrates the frequency dependence of the dielectric permittivity (Figure 7a) and the dielectric loss (Figure 7b) for HIPS + Si and HIPS + Si + MWCNT nanocomposites. The variation of the dielectric constant and dielectric loss of nanocomposites filled with Si, MWCNT hybrid systems within the investigated frequency range provides fundamental insight into the interaction of different polarization mechanisms in the composite system. The change in behavior from low to high frequencies is associated with both polymer chain dynamics and interfacial effects at the filler–polymer interface. Since the neat HIPS matrix contains weakly polar functional groups, it exhibits the lowest ε′ and ε″ values over the entire frequency range. This behavior is also related to the limited dipolar activity of the polymer chains. The incorporation of Si nanoparticles into the system leads to a significant increase in the dielectric constant. This enhancement is mainly attributed to the activation of Maxwell–Wagner–Sillars (MWS) interfacial polarization (Samet et al., 2022). The difference in electrical conductivity (Dil et al., 2020) and dielectric permittivity between the polymer matrix and Si particles results in charge accumulation at the interfaces at low frequencies, thereby increasing the overall polarization capability. As the concentration increases, particularly in the system containing 3% Si, a tendency toward particle agglomeration is observed, which may induce certain nonlinearity in the dielectric constant.

The hybrid incorporation of MWCNT and Si in the present system exerts a stronger influence on the dielectric properties of the composite and leads to the formation of the highest ε′ values. A similar increasing trend in dielectric properties has been reported by Altarawneh (2024). The siloxane–MWCNT nanocomposite’s dielectric characteristics improve as MWCNT concentration rises across the whole frequency range (Altarawneh, 2024).

The energy dissipation mechanisms in the composites are revealed by the frequency dependence of the dielectric loss. Based on the ε″(f) dependence, the effective relaxation time was calculated for the relaxation maximum recorded at 125 Hz for the samples (Kremer and Schönhals, 2003):

$$\tau =\frac{1}{2\pi f_{\max}}$$ (1)

It was determined that τ = 1.27 m. This relaxation time corresponds to interfacial (MWS-type) relaxation. As seen in Figure 7b, this relaxation peak is practically not observed for pure HIPS. It is also absent for HIPS+1%Si and HIPS+1.5%Si nanocomposites. Although the incorporation of Si generally increases the dielectric loss values, a distinct relaxation peak is not observed. The peak is recorded for the HIPS+3%Si, and it becomes sharply pronounced with the incorporation of MWCNT into the nanocomposite. The dielectric loss curves of HIPS + Si + MWCNT nanocomposites exhibit some fluctuations over the investigated frequency range. Such behavior can be attributed to the heterogeneous nature of the composites and the coexistence of several loss mechanisms. The conductive nature of MWCNTs leads to a significant increase in energy dissipation, charge relaxation at the nanotube–polymer and nanotube–Si interfaces, and the formation of near-percolation areas (Khan et al., 2020).

Around 200 kHz (logf≈5 on the x-axis), a weak secondary relaxation peak is observed for the HIPS + Si nanocomposites. Using Equation 1, the relaxation time calculated for this peak is τ ≈ 1.6 × 10^-6^s. This relaxation is attributed to secondary dipolar relaxation. In contrast, for HIPS+1%MWCNT+1%Si and HIPS + 1.5%MWCNT+1.5%Si systems, this relaxation is not observed around 200 kHz. This behavior is related to the conductive contribution of MWCNTs (Abdinov et al., 2025; Naghiyev et al., 2024) and is associated with dipolar high-frequency dispersion. Based on the ε″(f) dependence, the AC conductivity at 200 kHz was calculated for the HIPS+1.5%MWCNT+1.5%Si nanocomposite (see Equation 2) (Büyükbaş-Uluşan et al., 2020):

$$\sigma_{AC}={2\pi f\varepsilon }_{0}\varepsilon^{\prime \prime }$$ (2)

For the HIPS+1.5%MWCNT+1.5%Si nanocomposite, the AC conductivity value obtained at 200 kHz, σ_AC_ ≈ 10^−8^S/cm, is consistent with the results reported by Mergen et al. (2020). It should be noted that in their study, the authors obtained an AC conductivity value of σ_AC_ ≈ 10^−6^S/cm at 1 MHz for a polystyrene PS+16.3% MWCNT (Mergen et al., 2020). This difference can be attributed to the lower MWCNT content. It is known that electrical conductivity strongly depends on filler concentration near the percolation threshold in CNT-based polymer systems (Khan et al., 2020).

**FIGURE 8:**
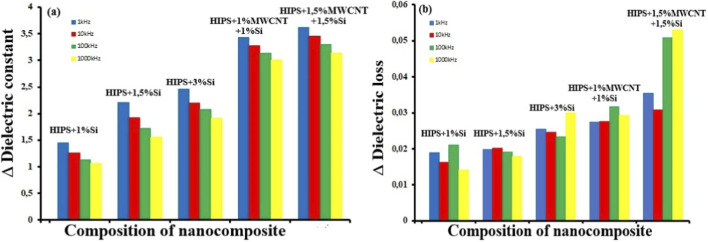
Composition-dependent variation of the **(a)** dielectric constant (Δε′) and **(b)** dielectric loss (Δε″) of HIPS-based nanocomposites at different frequencies in comparison with neat HIPS.

Figure 8 illustrates the composition-dependent variation of the dielectric constant (Δε′) (Figure 8a) and dielectric loss (Δε″) (Figure 8b) of HIPS-based nanocomposites at different frequencies in comparison with neat HIPS.

The largest change in terms of both dielectric constant and dielectric loss compared to neat HIPS is observed in the HIPS+1.5%MWCNT+1.5%Si nanocomposite, which is explained by the strong synergy created by the hybrid fillers, the near-percolation conductive network, and the intensive interfacial polarization mechanisms.

Yahya et al. (2020) investigated the percolation threshold of MWCNTs in a PVDF polymer matrix, which belongs to the class of thermoplastic polymers similar to HIPS, both theoretically and experimentally. It was reported that, from the viewpoint of dielectric properties, a concentration of about 1.5 wt% MWCNT determines the percolation threshold in the polymer (Yahya et al., 2020).

In the study presented by Staudinger et al. (2025), the percolation threshold for MWCNTs in a polymer matrix was also reported to be in the range of 1.five to two wt%. The formation of a conductive network in polymer/CNT composites can be explained by classical percolation theory (Staudinger et al., 2025).

Studies specifically addressing the determination of the percolation threshold in polymer systems filled with Si nanoparticles, particularly from the viewpoint of dielectric properties, are relatively limited. However, in the study by Zhao et al. (2021), molecular dynamics simulations were employed to investigate the formation of percolated networks of mixed nanoparticles of different sizes (small and large NPs) in polymer nanocomposites. The authors demonstrated that the percolation threshold is strongly influenced by the dispersion state, the maximum particle size, and the number of clusters formed in the system (Zhao et al., 2021).

Martínez et al. (2015) reported that, for carbon black particles with an average size of about 32 nm (comparable to the size of the Si nanoparticles used in the present study), the percolation threshold in a polystyrene matrix is approximately 10 wt% (Martínez et al., 2015).

However, when fillers with varying electrical characteristics, morphologies, and aspect ratios are present simultaneously in hybrid systems, the percolation behavior becomes more complicated. High-aspect-ratio conductive fillers, such MWCNTs, can create primary conductive channels in these systems at comparatively low concentrations. The creation of micro-capacitive structures and interfacial polarization are the primary functions of spherical or semiconductive particles like Si. The hybrid composite’s total percolation threshold may be lowered by the synergistic effect of these two filler types interacting.

The mechanical properties of the obtained nanocomposites were studied. The effect of Si and MWCNT additions on the parameters of HIPS polymer, such as tensile force, elongation, and relative elongation, is given in Table 1. It was found that as the composition of the films changes, the mechanical properties also change, which makes the mechanical behavior more complex and difficult to interpret. However, mechanical studies provide very rich information about the sample. Four different specimens (n = 4) of pure HIPS polymer were taken, and measurements were performed, showing that HIPS exhibits a relative elongation of up to 80% when no fillers are added. This is a result accepted in the literature (Knoeppel et al., 2014). With the addition of Si nanoparticles to HIPS polymer and increasing their concentration, an increase in the tensile strength value is observed. Accordingly, a decrease in the elongation value is observed.

**TABLE 1:** Mechanical properties of HIPS and HIPS-based nanocomposites.

Sample	n	Thickness (mm)	Tensile force (kg)	Tensile strength (MPa)	Elongation (mm)	Relative elongation (%)
HIPS	4	1.220 ± 0.014	11.40 ± 0.24	14.06 ± 0.43	20.50 ± 2.52	82.00 ± 10.07
HIPS + 1.5% Si	4	1.185 ± 0.021	13.03 ± 0.23	16.30 ± 0.09	6.38 ± 0.48	25.50 ± 1.91
HIPS + 3% Si	4	1.187 ± 0.022	13.38 ± 0.25	17.07 ± 0.19	5.00 ± 0.41	20.00 ± 1.63
HIPS + 1.5% Si + 1.5% MWCNT	4	1.387 ± 0.099	13.61 ± 0.18	17.49 ± 0.32	3.75 ± 0.65	17.88 ± 0.30

Changes in mechanical properties caused by the incorporation of high-hardness additives such as silicone into HIPS-type thermoplastic polymers have been investigated in similar studies. Moskalyuk et al. (2020) reported that adding small spherical SiO_2_ particles to polystyrene significantly increased the composite’s elastic modulus (Moskalyuk et al., 2020). However, Multi-walled carbon nanotubes are more recognized as high-performance reinforcements for polymers. Due to their extremely high aspect ratio and intrinsic strength, even a small fraction of well-dispersed MWCNT can dramatically improve the mechanical and electrical properties of a polymer composite (Yaqoob et al., 2025). Using silicon-based particles and MWCNT together in HIPS can potentially yield synergistic effects, combining the benefits of each nanofiller. Hybrid filler systems have been shown in various polymers to outperform single-filler composites by improving multiple properties simultaneously. Indeed, Ozbek and Cakir (2022) observed that combining MWCNTs with nano-silica had a “positive contribution” to the strength of adhesive joints, exceeding the effect of each filler by itself (Özbek and Çakır, 2022).

The observed trade-off between tensile strength and elongation can be attributed to the effects of filler dispersion and interfacial adhesion. Sharma et al. (2026) reported that, in polymer nanocomposites filled with nanoscale inorganic particles, the mechanical performance is primarily governed by the filler aspect ratio, percolation behavior, and interfacial adhesion with the polymer matrix. The filler–matrix interface and the associated interphase region are dominant contributors to composite behavior in nanoscale and hybrid systems. Strong interfacial bonding facilitates efficient stress transfer, and homogeneous dispersion of fillers is necessary for strength increase in polymer nanocomposites. Filler agglomeration results in decreased ductility and stress concentration areas. Since increasing filler loading limits matrix deformation and encourages premature failure, there has been widespread reporting of a constant trade-off between stiffness and elongation. The creation of filler networks in the polymer matrix, interphase properties, and dispersion quality are all closely correlated with these effects (Sharma et al., 2026).

## 4 Conclusion

The structural, morphological, dielectric, and mechanical characteristics of HIPS + Si and HIPS + Si + MWCNT nanocomposites were thoroughly examined in this work. The graphitic structure of MWCNTs in the nanocomposite and the polycrystalline nature of Si nanoparticles were verified by XRD studies. The surface morphology of the HIPS polymer was altered by the addition of nanofillers, as proved by AFM studies. t was found that the RMS surface roughness decreases from approximately 85 nm for pure MWCNTs to about 70 nm for HIPS + Si and further to around 30 nm for the HIPS + Si + MWCNT system, indicating refinement of structural elements. The obtained results confirm that improved dispersion of MWCNTs and the formation of a hierarchical hybrid structure in the ternary system lead to a more homogeneous surface morphology, which is consistent with the XRD findings. The HIPS + Si + MWCNT nanocomposites showed the highest dielectric constant and dielectric loss values, according to dielectric investigations. The dielectric loss increased from 0.0083 to 0.0612 at 1 MHz. Moreover, the dielectric constant of the HIPS+1.5 wt% Si+1.5 wt% MWCNT nanocomposite was approximately 3.5 times higher than that of pure HIPS, particularly at low frequencies (1 kHz).Strong interfacial polarization and the formation of a near-percolative micro-conductive network inside the matrix are responsible for this improvement. Examining the mechanical characteristics revealed that Si and MWCNT fillers reduced relative elongation while increasing tensile strength. These nanocomposites are promising options for advanced polymer applications where structural strength and functional response are vital, since the combination of Si and MWCNT fillers had a synergistic effect that improved both dielectric and mechanical performance.

## Data Availability

The original contributions presented in the study are included in the article/supplementary material, further inquiries can be directed to the corresponding authors.
